# The Soybean Purple Acid Phosphatase GmPAP14 Predominantly Enhances External Phytate Utilization in Plants

**DOI:** 10.3389/fpls.2018.00292

**Published:** 2018-03-12

**Authors:** Youbin Kong, Xihuan Li, Bing Wang, Wenlong Li, Hui Du, Caiying Zhang

**Affiliations:** North China Key Laboratory for Germplasm Resources of Education Ministry, Hebei Agricultural University, Baoding, China

**Keywords:** purple acid phosphatase, acid phosphatase, phytate, phosphorus utilization efficiency, soybean

## Abstract

Induction and secretion of acid phosphatases (APases) is considered to be an important strategy for improving plant growth under conditions of low inorganic phosphate (Pi). Purple acid phosphatases (PAPs), are an important class of plant APases that could be secreted into the rhizosphere to utilize organic phosphorus (Po) for plant growth and development. To date, only a few members of the PAP family have been identified in soybean. In this paper, we identified a secreted PAP in soybean, GmPAP14, and investigated its role in utilizing external phytate, the main form of organic phosphorus in the soil. An analysis of its expression and promoter showed that *GmPAP14* was mainly expressed in the root and was strongly induced following Po treatment, during which its expression expanded from meristematic to maturation zones and root hairs. *In vitro* enzyme assays indicated that GmPAP14 had a relatively high phytase activity. Furthermore, *GmPAP14* overexpression increased secreted APase activities and phytase activities, leading to the improved use of external plant phytate, higher phosphorus content, and increased shoot weight. Thus, these results confirmed that *GmPAP14* is an important gene induced in response to Po, and that it predominantly participates in utilizing external Po to enhance plant growth and development.

## Introduction

Phosphorus (P) is an essential macronutrient that plays an important role in plant growth and development (Marschner and Rimmington, 1988; Vance, 2001). However, in most soil, the content of inorganic phosphate (Pi), is much lower than that needed for plant growth. The majority of P exists in an organic form (Po) or is fixed with calcium, iron and aluminum, which cannot be directly utilized by plants (George and Richardson, 2008). Thereby, efficiency of P-utilization is a major target for improving crop productivity. In fact, to maintain or obtain high crop yields, phosphate fertilizers are commonly applied in farming; however, most phosphate fertilizers are not completely absorbed and become unavailable to crops. Additionally, large applications of fertilizers cause soil degradation and water eutrophication (Gilbert, 2009). Therefore, improving crop P acquisition and utilization efficiency is essential for sustainable gains in agricultural production (Song et al., 2014).

To survive in low Pi environments, plants have developed a wide range of adaptive responses to improve efficiency of P-utilization (Chiou and Lin, 2011; Wu et al., 2013), such as modification of root architecture (Devaiah et al., 2007), increased transport capabilities of high-affinity Pi transporters (Qin et al., 2012; Fiorilli et al., 2013; Liu et al., 2013), and increased acid phosphatase (APase) (Xiao et al., 2006; Hur et al., 2010; Liang et al., 2010). These evolutionary strategies are precisely controlled by different mediators in a complex P regulatory network. Of these various strategies, improvement of APase activity is considered that enables plants to utilize extracellular and intracellular Po (Kaida et al., 2010; Sun et al., 2013; Wang et al., 2014).

Purple acid phosphatase (PAP) is a type of APase and contains a binuclear metal ion center, which hydrolyses a wide range of phosphate esters and anhydrides under optimal acidic conditions. PAPs have five conserved motifs (**D**XG/G**D**XX**Y**/G**N**H(D/E)/VXX**H**/G**H**X**H**, bold letters represent invariant residues), which can coordinate the binuclear metal center to hydrolyse Po (Schenk et al., 1999; Olczak et al., 2003; Bozzo et al., 2004). Some of the biological functions of PAPs have been described including P acquisition and utilization (Ravichandran et al., 2013, 2015), peroxidation (Zhang et al., 2008), and response to salt tolerance (Liao et al., 2003; Li et al., 2008). Among these functions, P acquisition and utilization is widely studied and considered to be the most main research focus (Hurley et al., 2010; Kaida et al., 2010; Holme et al., 2016).

Multigene families of plant PAPs have been reported in *Arabidopsis* (Li et al., 2002), rice (Zhang et al., 2011), soybean (Li et al., 2012) and maize (Gonzalez-Munoz et al., 2015). Additionally, several of them have been studied and demonstrated to participate in Po utilization and mobilization. In *Arabidopsis, AtPAP26* was induced by Pi starvation, and its ability to utilize Po has been well characterized. *Atpap26*-mutant plants grew much smaller than the wild-type (WT) plants under a P starvation condition (Veljanovski et al., 2006; Hurley et al., 2010). *AtPAP10* is another APase gene that is induced during Pi starvation and plays an important role in plant tolerance to Pi limitation (Wang et al., 2011). In rice, *OsPAP10a* and *OsPAP10c* are induced by Pi starvation, and their over-expression increase APase activities and improve the utilization of external Po, suggesting that they are important APase genes in rice (Tian et al., 2012; Lu et al., 2016). In maize, 33 members of the PAP family have been identified, and an analysis of RNA-seq transcriptome data revealed that PAP genes were expressed in different plant tissues at multiple stages of development, suggesting they are generally important throughout the plant life cycle (Gonzalez-Munoz et al., 2015).

Soybean is an economically important nitrogen-fixing crop that is grown worldwide. Insufficient P is an important limiting factor for its growth and production (Song et al., 2014). Although PAPs are important enzymes for Po utilization that are generally induced in response to low Pi, the functions of the majority of PAPs remain unknown (Li et al., 2012). *GmPAP3*, the first documented PAP gene in soybean, is induced by NaCl stress, not by Pi starvation (Liao et al., 2003). *GmPAP4*, the second documented PAP gene, is induced by Po, and its overexpression enhanced Po utilization (Kong et al., 2014). *GmPAP21*, the third and most recently documented PAP gene, was induced in nodules, roots and old leaves, suggesting a potential role in P recycling in leaves and P metabolism in nodules (Li et al., 2017). In a previous study, we cloned a PAP gene in soybean, *GmPAP14*. It contained a 1395 bp encoding a polypeptide with 464 amino acid residues. A bioinformatics analysis predicted that GmPAP14 had the characteristics of an APase (Kong et al., 2012). In this paper, we have investigated GmPAP14 more thoroughly and demonstrated its roles in Po utilization.

## Materials and Methods

### Plant Materials and Growth Conditions

**FIGURE 1:**
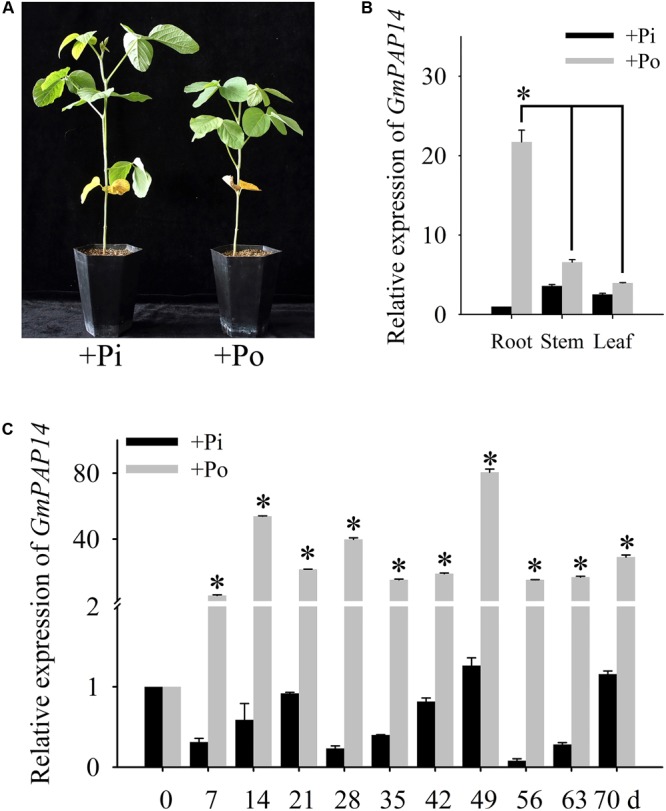
Analysis of *GmPAP14* expression patterns in soybean. **(A)** Phenotype of zhonghuang15 (ZH15) under KH_2_PO_4_ (+Pi) and Phytate (+Po). **(B)** Expression analysis of *GmPAP14* in different tissues of ZH15 under +Pi and +Po conditions. Seven-day-old seedlings were treated with +Pi and +Po. Seven days after treatment, roots, stems, and leaves were harvested for gene expression in different tissues. Asterisk represents significant difference of *GmPAP14* expression in root compared to that in leaf and stem under +Po conditions (*P <* 0.05, *t*-test). **(C)** Expression of *GmPAP14* in roots of ZH15. Seven-day-old seedlings were treated with +Pi and +Po (0 day was used as a control). Roots were sampled after 7, 14, 21, 28, 35, 42, 49, 56, 63, and 70 days and were used for temporal expression analysis. Asterisks represent significant differences of *GmPAP14* expression at the same growth period under +Pi and +Po conditions (*P <* 0.05, *t-*test). The relative expression value was calculated by the ratio of the expression value of *GmPAP14* to that of soybean housekeeping gene *GmActin11* using the 2^-ΔΔC_t_^ method. Each bar is the mean of three replicates with the standard error.

The soybean cultivar zhonghuang15 (ZH15, P-high efficiency, **Figure 1A**) and *Arabidopsis* (Columbia ecotype) were used in this study. The Pi and Po treatments in this study were performed using a modified Hoagland solution containing 22.3 μg/L MnSO_4_⋅4H_2_O, 0.83 μg/L KI, 0.025 μg/L CoCl_2_⋅6H_2_O, 8.6 μg/L ZnSO_4_⋅7H_2_O, 0.025 μg/L CuSO_4_⋅5H_2_O, 6.2 μg/L H_3_BO_3_, 0.25 μg/L NaMoO_4_⋅2H_2_O, 0.278 mg/L FeSO_4_⋅7H_2_O, 0.373 mg/L Na_2_EDTA, 0.505 g/L KNO_3_, 0.49 g/L MgSO_4_⋅7H_2_O, 1.18 g/L Ca(NO_3_)_2_⋅7H_2_O, supplemented with either 0.137 g/L KH_2_PO_4_ (1 mmol/L, Pi) or 0.11 g/L phytate (1 mmol/L, Po), and adjusted to a pH of 6.0.

### Quantitative RT-PCR

The seeds of ZH15 were surface-sterilized and placed in pots with vermiculite in a greenhouse under 16 h light (28°C) /8 h dark (24°C) cycles. After 7 days (d) of growth (0 d was used as a control), the seedlings were separately treated with Pi and Po. After another 7 days, roots, stems, and leaves were harvested to profile the expression of *GmPAP14* in different tissues. Roots were sampled after 7, 14, 21, 28, 35, 42, 49, 56, 63, and 70 days and were used for temporal gene expression profiling.

Total RNA was extracted using the RNAprep Pure Plant Kit (Tiangen, China). Then, the first-strand cDNA was synthesized with a PrimeScript^TM^ Reagent kit and the gDNA Eraser (Takara, Japan). Quantitative RT-PCR (qPCR) was performed with SYBR Green Mix (Takara, Japan) on a CFX96 (Bio-Rad, United States). *GmPAP14* primers (5′-TCAAGCAGCCCCTTCATTAG-3′ and 5′-AGTTTTCCTTCGGCAATCTTC-3′) and primers for the housekeeping gene *GmActin11* (5′-ATCTTGACTGAGCGTGG TTATTCC-3′ and 5′-GCTGGTCCTGGCTGTCTCC-3′) were used in qPCR (Wang et al., 2012). Relative expression was calculated using the 2^-ΔΔC_t_^ method (Livak and Schmittgen, 2001). The data are presented as the means of three biological replicates with SE.

### Biochemical Characterization of GmPAP14

The cDNA of *GmPAP14* was cloned into the *Eco*R I and *Hind* III sites of the pET32a vector and transformed in Transetta (DE3) chemically competent cells (TransGen Biotech, China). The primers used were 5′-GAATTCAAAGTTGAGAAGACAG-3′ and 5′-AAGCTTTTAATGTGAAACATGAG-3′. The transformed cells were induced with 0.6 mmol/L IPTG at 24°C for 12 h to produce the GmPAP14-His fusion protein. Recombinant GmPAP14 was purified using a His-Tagged Protein Purification Kit (CWBIO, China). APase and phytase activity measurements, thermal stability and the effect of pH on GmPAP14 activity were analyzed using the methods described by Kuang et al. (2009). All data are presented as the means of three replicates with SE.

### Vector Construction and Plant Transformation

To construct the overexpression vector, the full-length cDNA of *GmPAP14* was cloned and inserted into a pCHAC vector (containing an HA tag) that had been digested with *Bam*H I and *Kpn* I. The primers used for *GmPAP14* cDNA amplification were 5′-GGATCCATGGGTGTTGTGGAGGGTCTCT-3′ and 5′-GGTACCATGTGAAACATGAGCCGTGGAATCAT-3′. To construct the promoter vector, a 2568-bp *GmPAP14* promoter fragment was amplified by PCR using the primers 5′-AAGCTTAGTCGAAGATGGGGATGT-3′ and 5′-GGATCCTTTGCTCAAAACCCGTG-3′. The amplified fragment was cloned into the *Hind* III and *Bam*H I sites of the pBI121 vector in place of the *CaMV* 35S promoter. The reconstructed plasmids were transferred into *Agrobacterium tumefaciens* GV3101 using a freeze-thaw procedure and transgenic plants were produced via *Agrobacterium*-mediated floral dip (Clough and Bent, 1998).

### GUS Histochemical Analysis

The T_3_ *GmPAP14* promoter-*GUS* (*P*_*GmPAP14*_-*GUS*) transgenic plants were grown on agar under Pi and Po conditions. The roots were harvested for GUS staining after 15, 20, and 30 days, and the leaves were harvested for GUS staining after 15 days. The samples were immersed in a reaction buffer containing 50 mmol/L phosphate buffer (NaH_2_PO_4_ and Na_2_HPO_4_), pH 7.0, 10 mmol/L Na_2_EDTA, pH 8.0, 1 mmol/L K_4_Fe(CN)_6_⋅3H_2_O, 1 mmol/L K_3_[Fe(CN)_6_], 2 mmol/L X-Gluc, 0.1% Triton X-100, and incubated at 37°C overnight. After staining, the tissues were cleared with 70% ethanol. All the samples were observed and imaged using a BX51 microscope (Olympus, Japan).

### Western Blot Analysis of GmPAP14 Proteins

Western blotting was performed as described by Wang et al. (2011). The primary antibody [HA Tag monoclonal antibody (1:5000, Thermo Fisher, United States)], and the secondary antibody [goat anti-mouse IgG(H+L)-HRP (1:5000, Beijing Protein Innovation, China)] was used for the Western blot. The blotted membrane was detected using an Odyssey FC imaging system (LI-COR, United States).

### Secreted APases and Phytase Activity Measurements

To measure secreted APase activities, 15-day-old seedlings grown in the Pi condition were transferred to 2-mL Eppendorf tubes containing 1.5 mL of a liquid medium supplemented with 1 mmol/L ρ-NPP. After being held for 1 day at 24°C, 0.5 mL of 0.5 mmol/L NaOH was added to terminate the reaction. Absorbance was measured at 410 nm (Wang et al., 2009). APase activity was expressed as ρ-NP released per hour per plant. All experiments were repeated three times, with five plants per replicate.

To measure the secreted phytase activities, we transferred 15-day-old seedlings from the Pi condition to 2-mL Eppendorf tubes containing 1.5 mL of a liquid medium with 1 mmol/L phytate. After 1 day at 24°C. The liquid medium was incubated with 0.5 mL of malachite green reagent, then measured at 650 nm (Lu et al., 2016). Phytase activity was defined as Pi released per hour per plant. All experiments were repeated three times, with five plants per replicate.

For the quantitative analysis of APase and phytase activities in culture medium, transgenic and WT plants were sown on vermiculite under Pi and Po conditions. After 30 days of growth, the proteins were concentrated from the culture medium and measured using the previously described methods.

### Total P Measurement

Total P measurements were analyzed as previously described (Lu et al., 2016). First the samples were dried under 80°C to a constant weight. Then, the samples were digested in glass tubes with H_2_SO_4_ and H_2_O_2_ at 180°C. The digested solutions were cooled to room temperature, reacted with malachite green reagent, and measured at 650 nm.

### Photosynthesis Measurement

The transgenic and WT plants were grown under Pi and Po conditions. After 30 days of growth the carbon assimilation rates were measured using a CIRAS-3 portable photosynthesis system (PP Systems, United States).

### Statistical Analysis

All data were analyzed using SPSS 17.0 software (IBM, United States). Student’s *t*-test was used to identify differences between the observations.

## Results

### *GmPAP14* Was Strongly Induced in the Roots of Soybean Under the Organic Phosphorus Condition

Pi deficiency significantly inhibited soybean growth (**Figure 1A**), in the present study, the spatial and temporal expression of *GmPAP14* in response to Po was first investigated by qPCR. The results showed that *GmPAP14* expression in the roots was much higher than that in the leaves and stems, suggesting that *GmPAP14* was mainly expressed in the roots grown under the Po condition (**Figure 1B**). Subsequently, temporal expression of *GmPAP14* in the roots showed that *GmPAP14* was rapidly induced at 7 days post Po stress (DPP), and maintained high expression from 14 to 70 DPP, with the peak occurring at 49 DPP. In contrast, *GmPAP14* expression was very low and unaffected under the Pi condition (**Figure 1C**).

### The Promoter of *GmPAP14* Preferentially Drives Expression in *Arabidopsis* Roots

**FIGURE 2:**
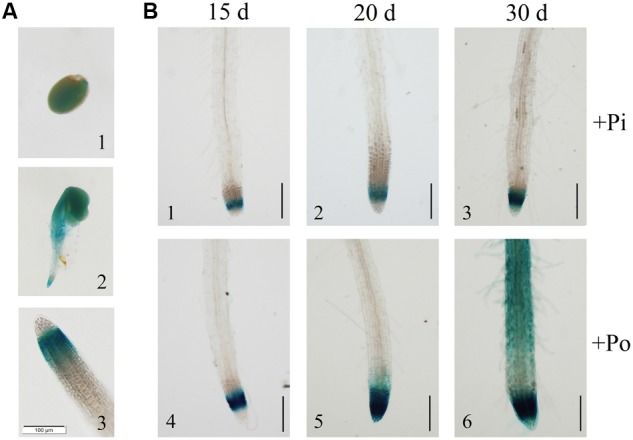
Analysis of the *GmPAP14* promoter in transgenic plants containing the *GmPAP14* promoter-GUS (*P*_GmPAP14_-*GUS*) reporter. **(A)** Histochemical localisation of GUS activity in transgenic plants **(1)** germinating seed. **(2)** 2-day-old seedling. **(3)** Root tip of 15-day-old seedling. The scale bar = 100 μm. **(B)** Analysis of the *GmPAP14* promoter response to the Po condition. The plants were sown in vermiculite and treated with KH_2_PO_4_ (+Pi) or Phytate (+Po). After 15, 20, and 30 days, the samples were harvested for GUS staining. The scale bar = 50 μm.

To further verify the expression of *GmPAP14*, a 2568-bp *GmPAP14* promoter sequence was cloned, fused to a GUS reporter gene (*P*_GmPAP*14*_-*GUS*) and transformed into WT *Arabidopsis* plants. First, we observed intense GUS staining in germinating seeds and cotyledons, and in the meristematic zones of the root during the early stages of seedling development (**Figures 2A1,A2**). In mature plants, GUS staining was predominantly detected in the root tips (**Figure 2A3**).

Subsequently, we analyzed the response of the *GmPAP14* promoter to low P at different developmental stages (**Figure 2B**). Stronger GUS staining was observed in the meristematic zone of the root 15 days after germination under the Po condition. As the plant developed, GUS staining was much stronger and expanded from the meristematic zone to the maturation zone and the root hairs under the Po condition. These results indicate that the *GmPAP14* promoter is induced, and its expression expands under the Po condition.

### Purification and Biochemical Characterization of the GmPAP14 Protein

GmPAP14 had all seven conserved amino acids required for its catalytic activity. To test its activity, recombinant 6 × His-GmPAP14 protein was purified by Ni-Agarose and detected on SDS–PAGE (**Supplementary Figure S1A**), which was further confirmed by immunoblotting with anti-His antibody (**Supplementary Figure S1B**). Enzymatic assays demonstrated that the pure GmPAP14 protein had APase activity with the highest activity at a pH of 7.5 (**Supplementary Figure S1C**). Additionally, GmPAP14 was found to be moderately thermostable with the highest activity at 35°C (**Supplementary Figure S1D**). An examination of its substrate specificity revealed that GmPAP14 had relatively high phytase activity (86% of APase activity, **Supplementary Figure S1E**).

### *GmPAP14* Overexpression Significantly Increased Activities of Secreted APase and Phytase

**FIGURE 3:**
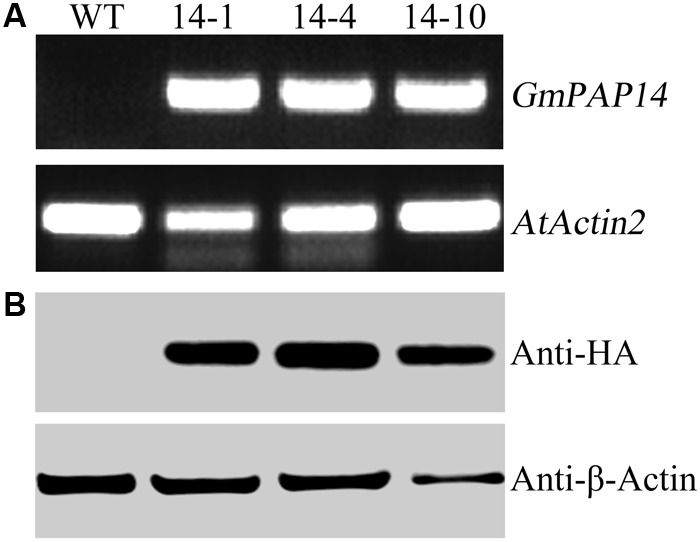
Analysis of *GmPAP4* transcripts and protein levels in transgenic plants. **(A)** Analysis of RT-PCR in wild-type (WT) and transgenic plants. **(B)** Western blot detection of GmPAP14 in WT and transgenic plants. RNA and protein were extracted from 30-day-old seedlings of transgenic lines 14-1, 14-4, and 14-10. A total of 1 μg of RNA was used for RT-PCR. A total of 6 μg of protein was loaded onto an SDS–PAGE gel. Separated proteins were transferred to a nitrocellulose filter membrane and probed with anti-HA proteins.

**FIGURE 4:**
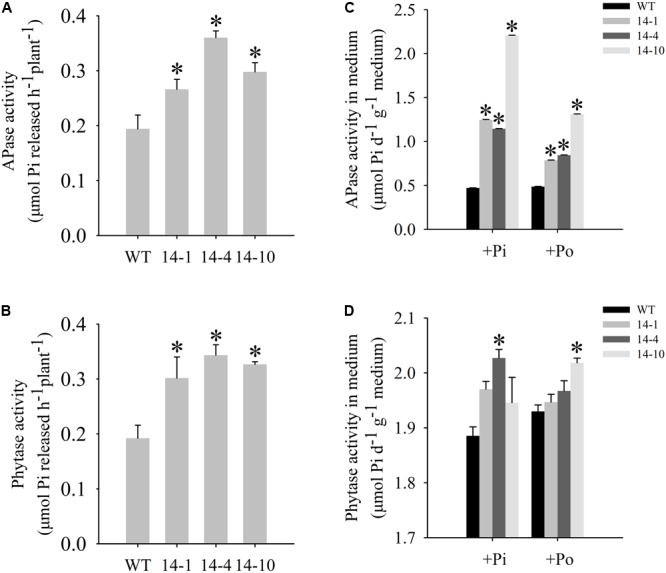
Measurement of secreted enzyme activities. **(A,B)** Secreted APase and phytase activities in the roots of transgenic plants (14-1, 14-4, and 14-10) and WT plants. 15-day-old seedlings from the Pi condition were transferred to 2-mL Eppendorf tubes containing 1.5 mL of a liquid medium supplemented with 1 mmol/L ρ-NPP or phytate. After being held for 1 day at 24°C, APase activity was measured at 410 nm. APase activity was expressed as ρ-NP released per hour per plant. Phytase activity was measured at 650 nm. Phytase activity was defined as Pi released per hour per plant. **(C,D)** APase and phytase activities in the medium of WT and transgenic plants. Transgenic and WT plants were sown on vermiculite under +Pi and +Po conditions. After 30 days of growth, the proteins were concentrated from the vermiculite, and enzyme activities were measured. The data are the mean ± SE (*n* = 5). Error bars represent the SE. Asterisks represent significant differences between transgenic and WT plants under the same condition (*P <* 0.05, *t-*test).

A bioinformatics analysis showed that GmPAP14 had a signal peptide sequence at its N terminal, indicating that it might be a secreted protein. To test this hypothesis and investigate the function of GmPAP14 in utilizing extracellular Po, the full-length cDNA of *GmPAP14* driven by the *CaMV* 35S promoter was transferred into *Arabidopsis*. Three independent transgenic lines expressing *GmPAP14* were obtained (**Figure 3A**). Immunoblotting also revealed that GmPAP14 proteins were highly and stably expressed in transgenic lines (**Figure 3B**). Subsequently, we measured the external enzyme activities of transgenic and WT plants. The results showed that the three independent transgenic lines had higher external APase activities than the WT plants (**Figure 4A**). Consistent with the higher APase activities, three *GmPAP14*-overexpressing lines also had higher external phytase activities than the WT plants (**Figure 4B**). These results suggested that *GmPAP14* overexpression could significantly increase secreted APase and phytase activities in *Arabidopsis*.

### *GmPAP14* Overexpression Significantly Increased Activities of APase and Phytase in Vermiculite

Next, we examined the APase and phytase activities of transgenic lines grown in vermiculite. The APase activity in vermiculite in which the transgenic plants were grown increased approximately 2–5 times under the Po condition compared to the activity in vermiculite in which WT plants were grown (**Figure 4C**). Phytase activity also increased in vermiculite in which the transgenic lines were grown (**Figure 4D**). These results add further support to GmPAP14 as a secreted protein and demonstrate its stability in vermiculite.

### *GmPAP14* Overexpression Significantly Increased Plant Biomass and P Content Under the Po Condition

**FIGURE 5:**
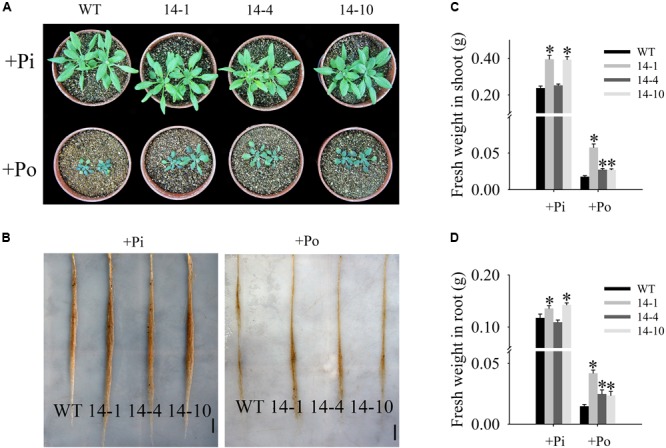
Measurement of transgenic and WT plant biomass under different phosphorus conditions. **(A,B)** Appearance of 30-day-old seedlings after treatment with KH_2_PO_4_ (+Pi) and Phytate (+Po). **(C,D)** Fresh weights of shoots and roots of transgenic and WT plants under +Pi and +Po conditions. The data are the mean ± SE (*n* = 5). Error bars represent the SE. Asterisks represent significant differences between transgenic and WT plants under the same condition (*P <* 0.05, *t-*test).

In this paper, the T_3_ transgenic and WT plants displayed different growth phenotypes under the Po condition. When Po was supplied, the three transgenic lines grew much better than the WT plants (**Figures 5A,B**) with shoot weight increases of 225.8, 54.3, and 52.8% (**Figure 5C**), and root weight increases of 183.9, 68.2, and 60.0% (**Figure 5D**). These results show that *GmPAP14* overexpression significantly increases plant biomass under the Po condition.

**FIGURE 6:**
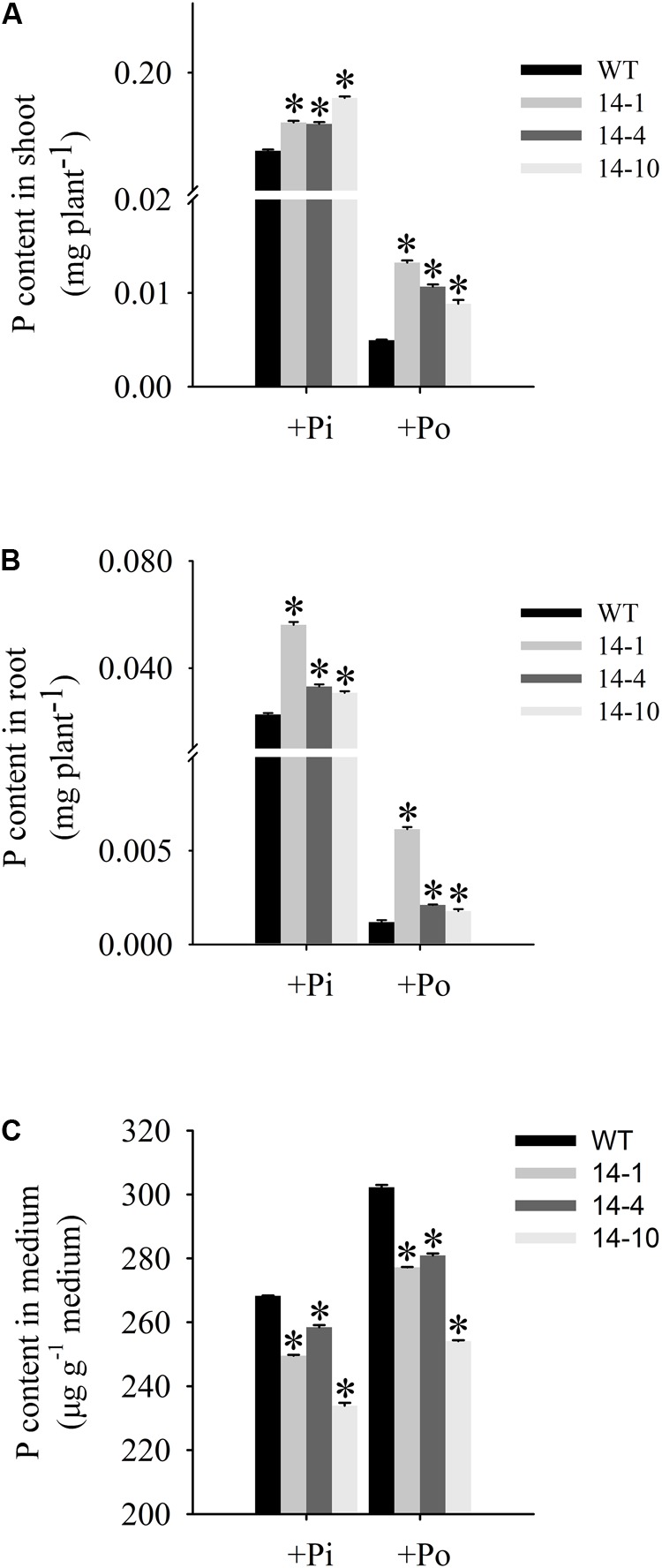
Organic phosphorus utilization efficiency of transgenic and WT plants under different phosphorus conditions. **(A)** P content in shoot. **(B)** P content in root. **(C)** P content in medium. After 30 days of growth, shoots, roots, and growth mediums were harvested separately for P content measurements. WT, wild-type plant. 14-1, 14-4, and 14-10: the three transgenic lines used. The data are the mean ± SE (*n* = 5). Error bars represent the SE. Asterisks represent significant differences between transgenic and WT plants under the same condition (*P <* 0.05, *t*-test).

An analysis of Po utilization revealed that P content increased in shoots and roots of *GmPAP14*-overexpressing plants under the Po condition compared to the WT plants (**Figures 6A,B**). Additionally, P content in vermiculite in which the transgenic plants were grown significantly decreased under the Po condition (**Figure 6C**).

### *GmPAP14* Overexpression Significantly Enhanced Photosynthesis in Plants Under the Po Condition

**FIGURE 7:**
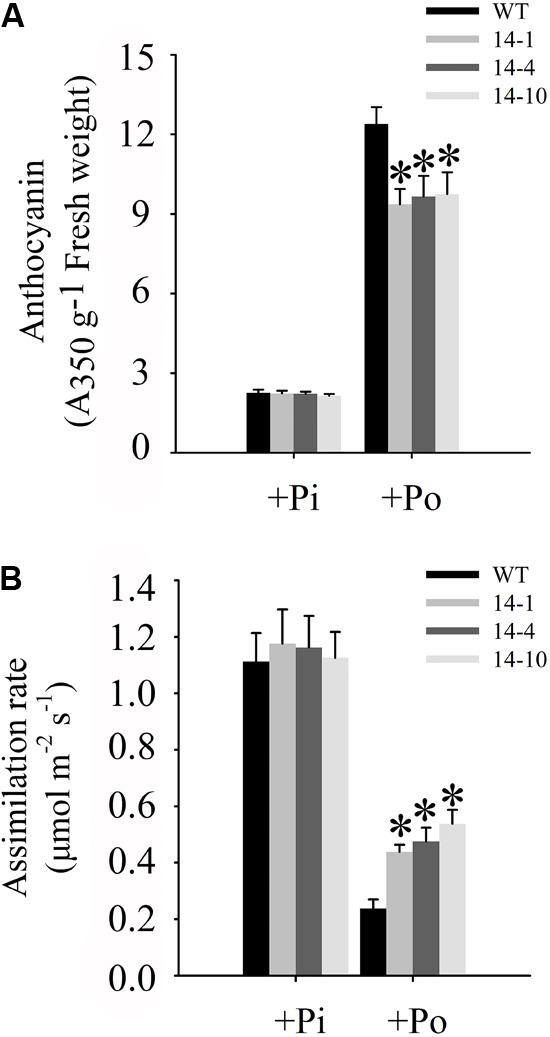
Measurements of photosynthetic efficiency in transgenic and WT plants under different phosphorus conditions. **(A)** Measurement of anthocyanin in transgenic lines (14-1, 14-4, and 14-10) and WT plants. **(B)** Assimilation rates of transgenic and WT plants were measured under +Pi and Po conditions. Transgenic and WT plants were sown on vermiculite under +Pi and +Po conditions. After 30 days of growth, leaves were harvested to measure anthocyanin and assimilation rates were measured using CIRAS-3. The data are the mean ± SE (*n* = 5 for **A**; *n* = 30 for **B**). The error bars represent the SE. Asterisks represent significant differences between transgenic and WT plants under the same condition (*P <* 0.05, *t-*test).

One of the obvious symptoms of plant Pi stress is anthocyanin accumulation in shoots, which is believed to protect chloroplasts from photoinhibition (Vance et al., 2003). Under the Po condition, anthocyanin concentrations in three independent transgenic lines were significantly lower than those in WT plants (**Figure 7A**). Compared to WT plants under the Po condition, measurements of carbon assimilation rates showed that *GmPAP14* overexpressing improved plant photosynthetic potential with 84.2, 100.0, and 126.3% increases in assimilation rates in the three independent transgenic lines (**Figure 7B**).

## Discussion

Acid phosphatases (EC 3.1.3.2) are considered important for plant responses to Pi starvation. Extracellular APases belong to a group of Pi starvation-inducible (PSI) phosphohydrolases secreted by roots (Tran et al., 2010). Secretion of PAPs, a specific group of PSI phosphohydrolases, has been shown to play an important role in external Po utilization (Wang et al., 2014). In this paper, *GmPAP14* was mainly expressed and induced in soybean roots under the Po condition (**Figure 1**), indicating that *GmPAP14* is a PSI gene that plays a crucial part in Po utilization. Moreover, GUS staining of transgenic plants with *P*_GmPAP*14*_-*GUS* indicated that the *GmPAP14* promoter not only was strongly induced but also extended its expression region from meristematic to maturation regions and root hairs (**Figure 2**). In our previous study, GmPAP14 signal peptides and the prediction of its subcellular localisation suggested that it was a secreted protein (Kong et al., 2012). In our current study, increases in the secreted APase activities of transgenic plants and APase activities in vermiculite further demonstrated that GmPAP14 could be produced in roots and secreted to facilitate the utilization of more Po around the roots (**Figures 4A,C**).

Purple acid phosphatases hydrolyse a wide range of organic P to produce Pi with an acidic pH optimum (Tran et al., 2010). Phytate is the main contributor of Po, accounting for approximately 60% of total soil Po (Wang et al., 2013). However, only a few studies have reported on the ability of PAPs to hydrolyse phytate. *AtPAP15* overexpression enhanced soybean plant growth on sand culture containing phytate (Kuang et al., 2009; Wang et al., 2009). Wang et al. (2011) reported that AtPAP10 had only moderate phytase activity (29% of APase activity), as determined by *in vitro* enzyme assays. In our previous work, we identified a soybean PAP gene, *GmPAP4*, which has phytase activity (45% of APase activity) and is involved in phytate hydrolysis and utilization (Kong et al., 2014). In this paper, we also found that GmPAP14 had characteristics of a phytase, and higher phytase activity (86% of APase activity). Evidence of GmPAP14’s phytase activity includes the following: (1) The significant induction of *GmPAP14* under the phytate condition (**Figure 1**) and its strong expression in germinating seeds (**Figure 2A**) and cotyledons (**Figure 2B**) suggested that it might scavenge phytate. (2) *In vitro* enzyme assays showed that GmPAP14 possessed a higher phytase activity (86%) relative to APase activity (**Supplementary Figure S1E**). (3) *GmPAP14* overexpression significantly improved secreted phytase activities of plants and phytase activities in vermiculite (**Figures 4B,D**). (4) Transgenic plants with *GmPAP14* overexpression obtained more P under the phytate condition compared to WT plants (**Figure 6**). Cumulatively, this evidence supports the conclusion that GmPAP14 is a phytase-like PAP, playing an important role in phytate utilization.

PAPs were suggested to act as multifunctional enzymes with roles in plant growth and development and peroxidase activity, but not in Po hydrolysis (Del Pozo et al., 1999; Zhu et al., 2005). For instance, histochemical staining of transgenic plants expressing an *AtPAP23* promoter-GUS reporter construct revealed that the transcription of *AtPAP23* was much stronger in flowers, suggesting that AtPAP23 may play an important role in flower development (Zhu et al., 2005). In our previous work, we found that transgenic plants with *35S*-*GmPAP4* had more lateral roots than WT plants (Kong et al., 2014). Lateral roots are initiated from pericycle founder cells (Tang et al., 2017). Later work with *GmPAP4* promoter-*GUS* plants also showed that GUS staining was detected in the stele (data not shown). This suggested that *GmPAP4* played a role in the formation of lateral roots. However, GmPAP14, another member of the PAP family in soybean, was mainly detected in the meristematic region of the root (**Figure 2**). Additionally, no significant differences in lateral root development were found in transgenic plants overexpressing *GmPAP14* (data not shown). Interestingly, GUS expression driven by a *GmPAP14* promoter was also detected in stomatal guard cells (**Supplementary Figure S2**), providing novel evidence of a role for *GmPAP14* in stomatal function. Although no direct support for our observations was found in the existing literature describing PAPs, several reports on *AtPHO1* described it as an Pi-induced gene that was primarily expressed in the root vascular tissues and played a role in the transfer of Pi (Hamburger et al., 2002; Rouached et al., 2011). Additionally, *AtPHO1* expression in guard cells influences stomatal closure in response to abscisic acid (ABA) in *Arabidopsis* (Zimmerli et al., 2012).

In summary, we have characterized the functions of *GmPAP14*. All our data clearly indicate that when plants suffer from low Pi stress, more GmPAP14 is mobilized and secreted from the root to utilize external Po. *GmPAP14* expression in stomatal guard cells may also influence their response to P starvation; however, additional experiments are needed to demonstrate this. Therefore, we infer that *GmPAP14* functions to improve efficiency of P-utilization and may also coordinate stomatal changes to promote photosynthesis under low-Pi conditions.

## Author Contributions

CZ, YK, and XL conceived and designed the research. YK and XL conducted the experiments and analyzed the data. BW, WL, and HD participated in parts of the experiments. YK drafted the manuscript. CZ revised the manuscript.

## Conflict of Interest Statement

The authors declare that the research was conducted in the absence of any commercial or financial relationships that could be construed as a potential conflict of interest.

## Supplementary Material

FIGURE S1 Biochemical characterization of GmPAP14. **(A)** SDS–PAGE analysis of purified GmPAP14. (1) protein molecular marker; (2) crude protein; (3) protein purified with Ni-agarose resin. **(B)** Immunoblot analysis of purified GmPAP14-His. (4) protein molecular marker; (5) GmPAP14-His hybridisation signal. **(C)** Thermal stability of GmPAP14. **(D)** pH response of GmPAP14 activity. **(E)** APase and phytase activities of purified GmPAP14. The data are the mean of three replicates with SE.

FIGURE S2 Analysis of *GmPAP14* promoter activity in leaves of transgenic plants. **(A)** Histochemical localisation of GUS activity in transgenic plants under the Pi condition. **(B)** Histochemical localisation of GUS activity in transgenic plants under the Po condition. The scale bar = 50 μm.
