# Supplementary figures and images for: The Soybean Purple Acid Phosphatase GmPAP14 Predominantly Enhances External Phytate Utilization in Plants
Source: Front Plant Sci. 2018 Mar 12;9:292. doi: 10.3389/fpls.2018.00292 (PMC5857590; doi:10.3389/fpls.2018.00292)

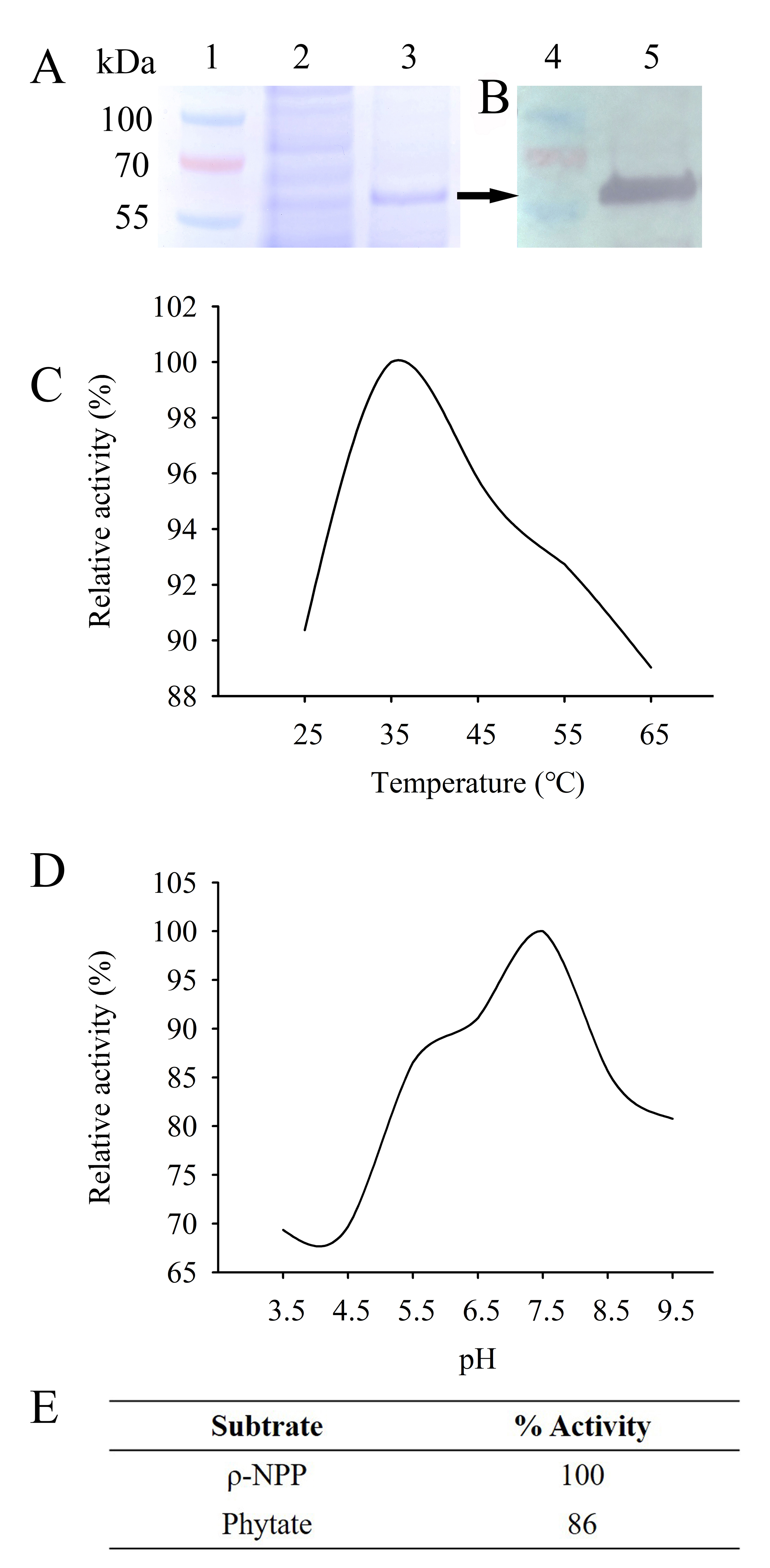

Supplement: FIGURE S1 — Biochemical characterization of GmPAP14. (A) SDS–PAGE analysis of purified GmPAP14. (1) protein molecular marker; (2) crude protein; (3) protein purified with Ni-agarose resin. (B) Immunoblot analysis of purified GmPAP14-His. (4) protein molecular marker; (5) GmPAP14-His hybridisation signal. (C) Thermal stability of GmPAP14. (D) pH response of GmPAP14 activity. (E) APase and phytase activities of purified GmPAP14. The data are the mean of three replicates with SE. [file Image_1.JPEG]

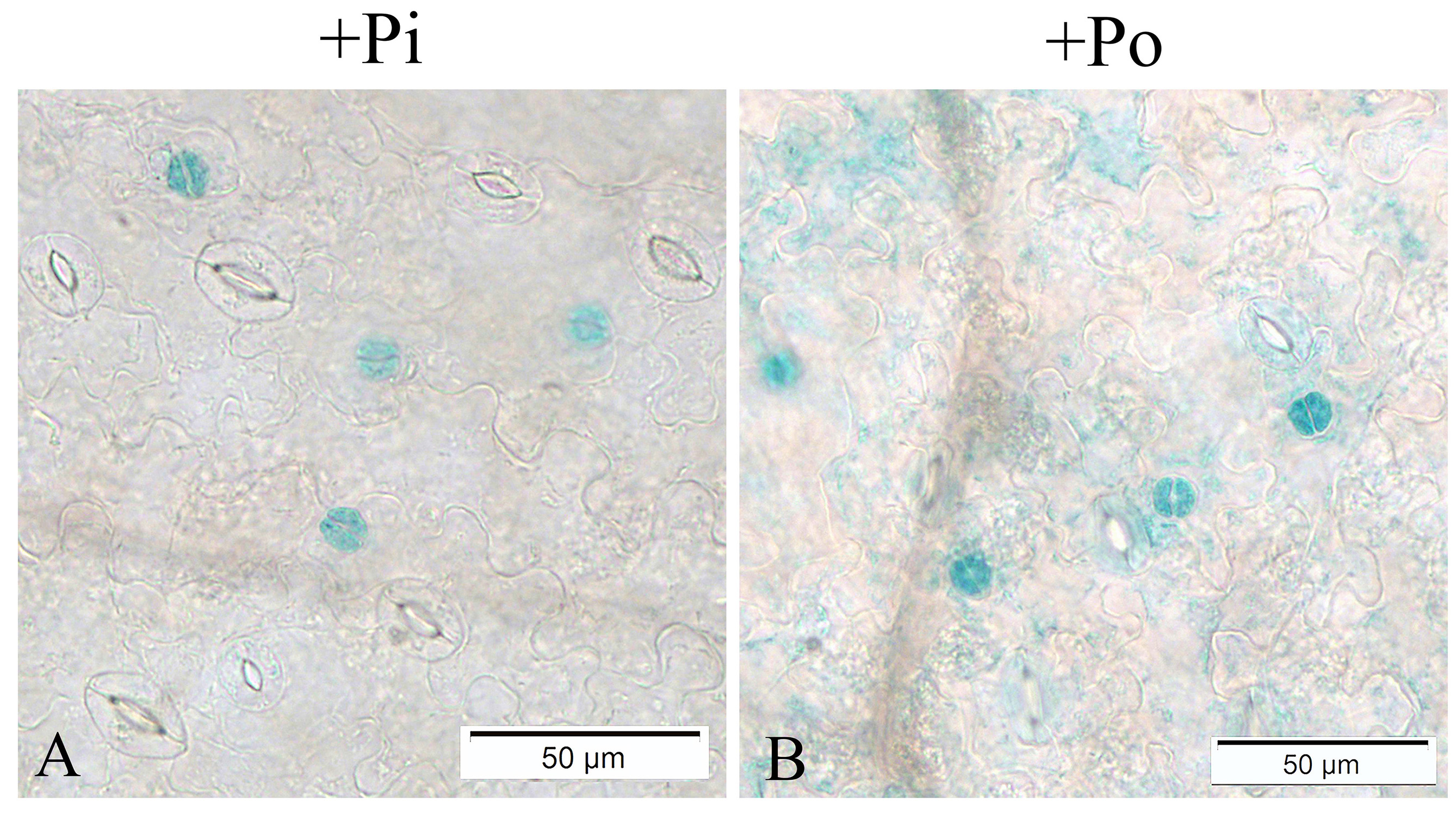

Supplement: FIGURE S2 — Analysis of GmPAP14 promoter activity in leaves of transgenic plants. (A) Histochemical localisation of GUS activity in transgenic plants under the Pi condition. (B) Histochemical localisation of GUS activity in transgenic plants under the Po condition. The scale bar = 50 μm. [file Image_2.JPEG]
